# Evaluating patient values and preferences for thromboprophylaxis decision making during pregnancy: a study protocol

**DOI:** 10.1186/1471-2393-12-40

**Published:** 2012-05-30

**Authors:** Pablo Alonso-Coello, Shanil Ebrahim, Gordon H Guyatt, Kari AO Tikkinen, Mark H Eckman, Ignacio Neumann, Sarah D McDonald, Elie A Akl, Shannon M Bates

**Affiliations:** 1 Iberoamerican Cochrane Centre, CIBERESP-IIB Sant Pau, Barcelona, 08041, Spain; 2 Department of Clinical Epidemiology and Biostatistics, McMaster University, Hamilton, ON, Canada; 3 Department of Medicine, McMaster University, Hamilton, ON, Canada; 4 Department of Urology, Helsinki University Central Hospital and University of Helsinki, Helsinki, Finland; 5 Division of General Internal Medicine and Center for Clinical Effectiveness, University of Cincinnati, Cincinnati, OH, USA; 6 Department of Internal Medicine, Pontificia Universidad Católica de Chile, Santiago, Chile; 7 Departments of Obstetrics & Gynecology, Radiology, and Clinical Epidemiology and Biostatistics, McMaster University, Hamilton, ON, Canada; 8 Department of Medicine, State University of New York at Buffalo, New York, NY, USA; 9 Thrombosis and Atherosclerosis Research Institute, Hamilton, ON, Canada

## Abstract

**Background:**

Pregnant women with prior venous thromboembolism (VTE) are at risk of recurrence. Low molecular weight heparin (LWMH) reduces the risk of pregnancy-related VTE. LMWH prophylaxis is, however, inconvenient, uncomfortable, costly, medicalizes pregnancy, and may be associated with increased risks of obstetrical bleeding. Further, there is uncertainty in the estimates of both the baseline risk of pregnancy-related recurrent VTE and the effects of antepartum LMWH prophylaxis. The values and treatment preferences of pregnant women, crucial when making recommendations for prophylaxis, are currently unknown. The objective of this study is to address this gap in knowledge.

**Methods:**

We will perform a multi-center cross-sectional interview study in Canada, USA, Norway and Finland. The study population will consist of 100 women with a history of lower extremity deep vein thrombosis (DVT) or pulmonary embolism (PE), and who are either pregnant, planning pregnancy, or may in the future consider pregnancy (women between 18 and 45 years). We will exclude individuals who are on full dose anticoagulation or thromboprophylaxis, who have undergone surgical sterilization, or whose partners have undergone vasectomy. We will determine each participant's willingness to receive LMWH prophylaxis during pregnancy through direct choice exercises based on real life and hypothetical scenarios, preference-elicitation using a visual analog scale (“feeling thermometer”), and a probability trade-off exercise. The primary outcome will be the minimum reduction (threshold) in VTE risk at which women change from declining to accepting LMWH prophylaxis. We will explore possible determinants of this choice, including educational attainment, the characteristics of the women’s prior VTE, and prior experience with LMWH. We will determine the utilities that women place on the burden of LMWH prophylaxis, pregnancy-related DVT, pregnancy-related PE and pregnancy-related hemorrhage. We will generate a “personalized decision analysis” using participants’ utilities and their personalized risk of recurrent VTE as inputs to a decision analytic model. We will compare the personalized decision analysis to the participant’s stated choice.

**Discussion:**

The preferences of pregnant women at risk of VTE with respect to the use of antithrombotic therapy remain unexplored. This research will provide explicit, quantitative expressions of women's valuations of health states related to recurrent VTE and its prevention with LMWH. This information will be crucial for both guideline developers and for clinicians.

## Background

Pregnancy-associated venous thromboembolism (VTE), which may manifest as pulmonary embolism (PE) or deep vein thrombosis (DVT), is an important cause of maternal morbidity [1]. PE remains the leading cause of mortality in developed countries, accounting for approximately 30% of all maternal deaths [2-5].

Although cohort studies have consistently demonstrated that pregnant women with prior VTE are at increased risk of recurrence [6], they have varied considerably in their estimates of magnitude of risk [7-11]. In the largest prospective study of 125 pregnant women with a single previous episode of objectively diagnosed VTE in whom antepartum heparin was withheld [9], the incidence of antepartum recurrence was 2.4% (95% CI of 0.2 to 6.9%). In subsequently published large retrospective cohort studies, the probability of antepartum VTE in women not given antepartum prophylaxis was approximately 6% [12,13]. The inclusion of women with more than one prior episode of VTE as well as women with pregnancies ending in loss, and the failure to independently adjudicate recurrent events might account for the higher risk of recurrence observed in these retrospective studies. Despite the inconsistency, the overall risk of antepartum recurrent VTE in all studies was less than 10% and confidence intervals around the risk estimates of individual studies were overlapping.

The risk of recurrent VTE in the non-pregnant population is lowest among women whose thrombosis was provoked by a major transient risk factor, intermediate among those with an associated minor reversible risk factor, and highest among those whose thrombosis was provoked by a persistent risk factor or who had an unprovoked event [14-30]. Although thrombophilic abnormalities are risk factors for a first episode of VTE [19], these abnormalities do not appear to play an important role in the risk of recurrence [14,15,19,20,30-40]. Data regarding predictive factors for recurrent VTE during pregnancy are inconsistent and studies have not found a clear association between the presence or absence of transient risk factors or of a definable thrombophilia and the risk of recurrent VTE associated with pregnancy [12,13].

Providing thromboprophylaxis to those women at increased risk of thrombosis can potentially reduce pregnancy-related recurrent VTE. However, prophylaxis during pregnancy is problematic. Warfarin crosses the placenta and has the potential to cause teratogenicity and bleeding in the fetus [41-44]. Prophylactic LMWH does not cross the placenta [45] or increase the risk of serious adverse fetal outcomes [44-54], and does not appear to increase the risk of heparin-induced thrombocytopenia (<0.1%) or heparin-associated osteoporosis (<1%) [46,47,51,55-68]. However, prophylactic LMWH is expensive, inconvenient, uncomfortable to administer, may be associated with an increased risk of major obstetrical bleeding [46], and generally necessitates a planned delivery to permit epidural analgesia [46]. Additionally, women may perceive that LMWH creates an undesirable “medicalization” of their pregnancy.

A Cochrane systematic review [69] identified two randomized trials comparing heparin prophylaxis to placebo or no prophylaxis in pregnant women with prior VTE [11,70]. Both suffered from major methodological weaknesses including very small sample sizes. Current clinical guidelines are based primarily on the observational studies described above and indirect evidence suggesting that LMWH substantially decreases the risk of VTE by approximately 70% in a wide variety of clinical contexts [46,71].

In considering women’s choices of thromboprophylaxis during pregnancy, two considerations are of particular importance. First, treatment decisions during pregnancy have implications not only for the health of the mother, but also for the health of the fetus. Second, many women prefer to see pregnancy as a normal part of a healthy woman’s life, rather than as a medical condition. Additional considerations include the burden associated with the management: frequency and route of administration, pain, discomfort, and possible side effects of the medication, and the need, frequency and type of testing associated with a given regimen.

Given that the desirable consequences do not clearly outweigh the undesirable consequences of prophylaxis (or vice versa), the values and preferences of pregnant women should be taken into consideration when making management decisions. Because individuals have different attitudes toward risk, the uncertainty of estimates for both the baseline risk of recurrent VTE and the effects of thromboprophylaxis makes the consideration of individual preferences even more important.

There are two fundamental approaches to “patient-specific” decision-making: (1) a holistic direct choice procedure and (2) utility elicitation from individual patients followed by “patient-specific” decision analysis. The relative merit of these approaches is open to question and few studies have addressed this issue [72]. In the “direct choice” methods, participants are presented with relevant health states and the probabilities associated with occurrence of those health states under alternative management strategies. The most rigorous and widely used experimental direct choice method is the probability trade-off or probabilistic version of the threshold technique and it involves determining the threshold benefit at which patients accept a treatment with fixed undesirable consequences or the threshold toxicity or adverse effects at which patients will decline a treatment with fixed benefit [73,74]. Investigators can also use other simpler direct choice methods (e.g. decision board) that provide complementary information and can ensure understanding of the probability trade-off.

The second approach to decision-making involves decision analytic modeling using best estimates of the probability of occurrence of relevant health states under the management options being considered. Patients’ ratings of the disutility associated with relevant health states (typically on scales that range from death to full health) then inform the decision model and allow calculations of the quality adjusted life years associated with management options. There are a number of approaches available for eliciting health state evaluations [75]. The standard gamble approach is most consistent with utility theory and is generally preferred by health economists [76,77]. Although the visual analog scale is theoretically less satisfactory than the standard gamble, it is much easier to understand, takes far less time to administer, and has superior psychometric measurement properties than to the standard gamble [78-82].

In atrial fibrillation [83,84], and to a lesser extent in VTE and thrombolytic therapy for stroke [85], investigators have studied patients' values and preferences. A recently completed systematic review of patient preferences for antithrombotic treatment did not identify any studies of pregnant women [86]. The objective of this study is to address this gap in knowledge.

## Methods

We will perform a multi-center international cross-sectional interview study at six centers in four countries: two in Hamilton, Ontario, Canada; one in Buffalo, USA; one in Oslo, Norway; and two in Helsinki, Finland.

### Study population and eligibility criteria

Pregnant women with a history of lower extremity DVT or PE who are considering thromboprophylaxis to prevent recurrent antepartum VTE constitute the ideal patient population. However, only a small number of such women are expected to present at the six centers over the time frame of the study. Therefore, we will also include women with a history of lower extremity DVT or PE who are planning pregnancy, and women who are 18 to 45 years of age with a history of lower extremity DVT or PE and may at some point be planning a future pregnancy. We will exclude women who are currently receiving thromboprophylaxis or full-dose anticoagulation, have undergone surgical sterilization (tubal ligation or hysterectomy), have a partner who has had a vasectomy, and those unwilling or unable to provide informed consent.

### Recruitment strategy

We will prospectively identify women who are currently pregnant or planning a pregnancy as they are referred for counseling, and identify women with a history of VTE with the potential to become pregnant by reviewing patient files. We will approach women referred for consideration of thromboprophylaxis prior to their consultation and will make initial contact with women who are not currently pregnant or planning a pregnancy by letter and then by telephone.

### Study maneuvers

A member of the research team will meet with women who express an interest in participating, and will explain the purpose of the study and carry out the informed consent process. If patients decline to participate, we will collect the reason for refusal. If patient consent to the study, we will make arrangements for interviews. We will use standardized scripts developed by the research team to ensure a standard approach across centres and interviewers. Expert and non-expert clinicians and allied health professionals have reviewed and revised the scripts to ensure understandability and readability by a lay person with a grade 9 reading level.

#### The participant interview

**Figure 1:**
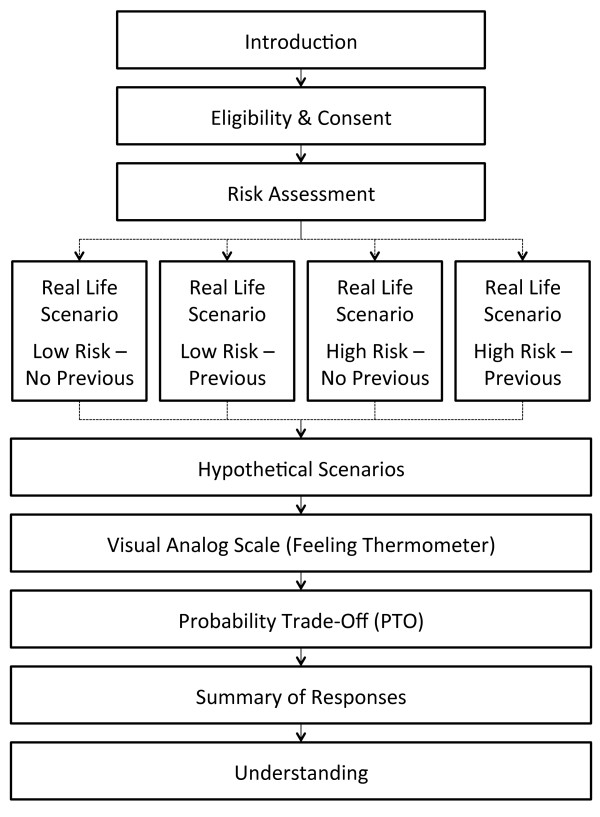
Interview flow chart.

Figure 1 presents the flow of the interview. We will determine patients' age, educational attainment, and current pregnancy status, as well as specific details about their past venous thromboembolic events (including occurrence of PE or DVT, number of events, date of the last event, presence or absence of precipitating risk factors prior to their event [Table 1]), known hypercoagulable states (Table 1), family history of VTE, type and duration of treatment for their event(s), adverse treatment effects, completeness of their recovery (for example, presence or absence of residual chest pain or shortness of breath, and/or residual leg swelling, pain or discoloration), and presence or absence of prior experience with injection of prophylactic doses of LMWH during pregnancy.

**Table 1:** Potential precipitating risk factors assessed regarding the past venous thromboembolic event Minor transient (i.e. now resolved) risk factors within 8 weeks of initial VTE

**Minor transient (i.e. now resolved) risk factors within 8 weeks of initial VTE**	**Major transient (i.e. now resolved) risk factors within 8 weeks of initial VTE**	**Known hypercoagulable state**
Pregnancy	Leg casting	Deficiency of antithrombin, protein C, or protein S
Postpartum (defined as 6 weeks after delivery)	Major surgery (>30 minutes; general or spinal anesthesia)	Activated protein C resistance/factor V Leiden
Hormonal contraception (birth control pill, patch or needle)	Acute medical illness with hospital admission for ≥ 3 days	Prothrombin gene mutation
Airplane travel (longer than 6 hours)	Immobilization ≥ 3 days (in bed except to go to washroom)	Anticardiolipin antibody positivity
	Active cancer	Nonspecific inhibitor

#### Direct choice exercises

We will determine each participant’s willingness to receive LMWH prophylaxis through direct choice exercises. Women will initially complete what we refer to as the real-life scenario, followed in order by hypothetical scenarios, the visual analog scale, the probability trade-off exercise, a review of their answers, and finally questions to examine their understanding their understanding of the scenarios. Use of decision boards will facilitate patient understanding of the direct choice exercises. Descriptions of each phase of the interview follow.

##### Real life scenario

We will initially present each woman with a decision board that will include the probabilities of developing VTE during pregnancy given the characteristics of her prior VTE (see below). This scenario will contain information similar to that received during standard clinical care but presented in a more systematic manner than that is typical in clinical practice.

**Figure 2:**
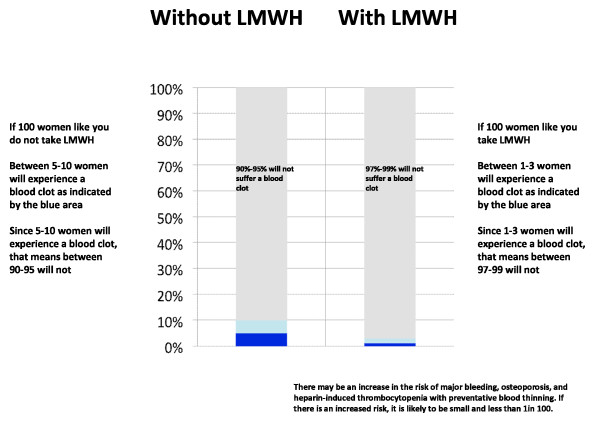
Example of bar chart presentation for women considered at high risk of recurrent VTE during pregnancy.

**Figure 3:**
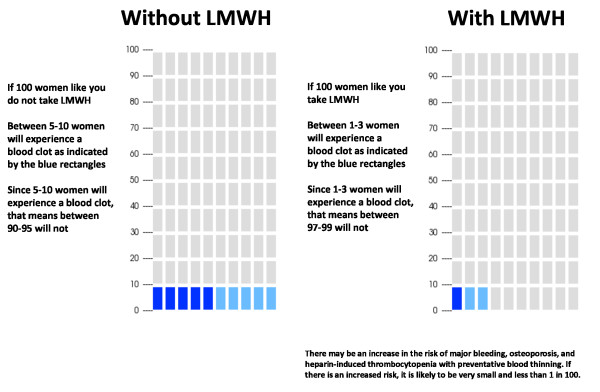
Example of pictogram presentation for women considered at high risk of recurrent VTE during pregnancy.

For this real life exercise, we will have two potential scenarios. We will classify women as low or high risk of recurrence. We define low risk of recurrence as the absence of a known thrombophilia and prior VTE associated with a major transient risk factor within 8 weeks prior to their last event, and higher risk of recurrence as prior unprovoked VTE, VTE associated with a minor transient risk factor within 8 weeks prior to the event or any VTE in association with a known hypercoagulable state. (Table 1) We estimate that the risk of antepartum recurrence for women judged to be at lower risk lies between 0 and 5%, while that for higher risk women ranges between 5 and 10%. We will assume that prophylactic LMWH reduces the risk of antepartum recurrence by approximately 70% [71]. To ensure optimal understanding, we will present the risk of recurrence with and without LMWH prophylaxis in three different ways: table, bar chart and pictograph (Table 2 and Figures 2 and 3). 

**Table 2:** Example of table presenting the risk of antepartum VTE recurrence for women considered at high risk of recurrent VTE during pregnancy

	**Without low molecular weight heparin use**	**With low molecular weight heparin use**
Probability of developing a blood clot during your pregnancy	5-10 in 100	1-3 in 100
Probability of NOT developing a blood clot during pregnancy	90-95 in 100	97-99 in 100

For the VTE health state, we will instruct women to consider their previous venous thromboembolic event. Women with more than one event will consider their most recent episode. We will instruct women with previous experience in the use of prophylactic LMWH for longer than 2 weeks during pregnancy to consider their previous experience when making a decision. We have prepared a description of the experience of LMWH use throughout pregnancy for women without experience in the use of LMWH prophylaxis during pregnancy (Additional file 1: Appendix 1). To ensure clinical verisimilitude, clinicians with considerable experience in the use of LMWH in pregnant women prepared the description and experts in obstetrics and thromboembolism reviewed them.

After reviewing this information, we will ask participants to decide whether or not they are willing to take LMWH during their pregnancy (for those who are pregnant) or whether they would be willing to do so in a subsequent pregnancy. Women currently pregnant and those referred for consideration of prophylaxis with a future pregnancy will meet with their health care provider and may wish to discuss the information provided with other people (for example, family members, friends, family physician, midwife or obstetrician). We will record the final decision after these additional consultations.

##### Hypothetical scenarios

We will provide study participants with three standardized scenarios in which the baseline risk of recurrent VTE, with and without the use of LMWH (assuming a 70% relative risk reduction) [71], will be varied (Table 3). Interviewers will show each woman a decision board with pictograms representing a low, followed by a medium, then a high risk of recurrent VTE. Participants will express their willingness to use LMWH given the associated burden (either based on their own prior experience or the standardized description [Additional file 1: Appendix I]), and the absolute magnitude of VTE risk reduction associated with the varying VTE recurrence risks. 

**Table 3:** Hypothetical scenarios with variable baseline risks of recurrent VTE and estimates of LMWH effectiveness

	**Risk of recurrence**		
	**Low risk**	**Medium risk**	**High risk**
Without LMWH	4 in 100	10 in 100	16 in 100
	(96 in 100 will not)	(10 in 100 will not)	(84 in 100 will not)
With LMWH	1 in 100	3 in 100	5 in 100
	(99 in 100 will not)	(97 in 100 will not)	(95 in 100 will not)

##### Probability trade-off

Interviewers will undertake probability trade-off exercises with "ping-ponging" to determine participant thresholds for accepting LMWH prophylaxis [73,74]. We will use a scenario based on the absolute effects of LMWH versus no prophylaxis for prevention of recurrent VTE during nine months of pregnancy. The interviewer will systematically vary the risk of VTE with LMWH prophylaxis (alternating between high and low risks) to determine the minimum acceptable reduction in the risk of VTE with prophylaxis at which the participant would agree to initiate LMWH. Based on the 95% CI surrounding the incidence of antepartum VTE in high risk patients reported in the prospective cohort study [9], we estimate that the upper bound of risk of recurrent VTE is 16 out of 100 without prophylaxis. Therefore, we will set this risk fixed on one side of the flipchart and will start offering probabilities ranging from 16 fewer VTE events per 100 pregnancies (maximum absolute risk reduction) to 0 less VTE events (same VTE risk as no prophylaxis) on the other side of the chart.

##### Visual analog scale (feeling thermometer)

Interviewers will determine the value patients place in relevant health states using a visual analog scale called the Feeling Thermometer (FT) [75]. When making ratings using the FT, women choose the score on the thermometer that represents the value they place on the health state they are evaluating. The FT is anchored at death (0) and full health (100). We will ask participants to consider (i) the health state of pregnancy with LMWH prophylaxis using the standard description or their previous experience (for those with two weeks or more of prophylactic LMWH during pregnancy), (ii) a pregnancy with their own most recent VTE experience, (iii) a standardized health state with a pregnancy-related DVT, (iv) a standardized health state with a pregnancy-related PE and (v) a standardized health state representing an obstetrical bleed (Additional file 1: Appendix I).

#### Check for consistency and understanding

After presenting the descriptions and recording patient responses, interviewers will review participant responses to the various exercises to check for consistency in the participant’s choice. When interviewers identify inconsistencies, they will offer participants a chance to review and change their responses, avoiding any suggestion that responses should be changed. The reasons for any apparent inconsistencies will be determined and recorded. Following this consistency check, interviewers will ask participants two standardized questions to evaluate their understanding of the information provided during the interview [Additional file 1: Appendix II]. Interviewers will also provide a rating of the extent to which they believe the respondents had a clear understanding of the questions and their confidence in this assessment.

### Outcomes

The primary outcome measure will be the minimum threshold reduction in VTE risk in the probability trade-off exercise at which women switch from declining to accepting LMWH prophylaxis (which we will refer to as the “VTE threshold”).

Secondary outcomes will include:

(i) women’s willingness to take prophylactic LMWH according to their classification as either high or low risk for recurrence,

(ii) women’s willingness to take prophylactic LMWH for each of the three hypothetical scenarios

(iii) Utilities for each of the five health states assessed in the FT (the burden of prophylactic LMWH use during pregnancy – either the standardized experience or the patient’s prior experience-, a pregnancy with the participant’s own most recent VTE, a standardized description of pregnancy-related DVT, a standardized description of pregnancy-related PE, and a standardized description of an obstetrical bleed [Additional file 1: Appendix I]).

**Figure 4:**
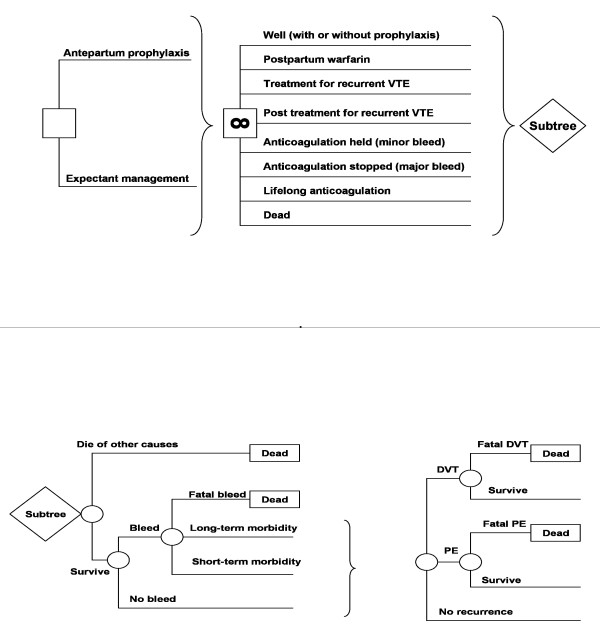
**Markov state transition decision model used in analysis**. This diagram depicts the model used in the analysis. In the figure on the top, the square on the left represents a “decision node” from which 2 branches, representing alternative management strategies emanate. Each strategy leads to the same Markov node, represented by a square with the “∞” symbol. The branches leading from the Markov node represent the various potential health states that patients pass through during the model simulation. Although the potential states for each strategy are the same, the initial distribution among states and probabilities associated with transitions between states will differ between strategies. The figure on the bottom is the modeled adverse events. During each time period or “cycle”, modeled patients are at risk for various adverse events. Round nodes represent the chance events. For each patient, the sequence of outcomes at these “chance” nodes will determine the state at which they begin the next cycle.

### Analysis

#### Baseline characteristics

We will describe age, educational attainment, pregnancy status, number and characteristics of previous venous thromboembolic events, presence or absence of precipitating risk factors, and prior experience with prophylactic LMWH injections using means and standard deviations or proportions, as appropriate.

#### Primary and secondary outcome(s)

We will calculate the mean threshold reduction in VTE at which women were willing to accept use of LMWH and the 95% confidence interval around the mean. We will calculate the proportion of women who are willing to take prophylactic LMWH and the associated 95% confidence intervals The mean and standard deviation of the visual analog scale ratings for each health state will also be determined.

Multiple variable linear regression will be undertaken to explore determinants of the VTE threshold, as determined in the probability trade-off exercise. In this analysis, VTE threshold will be the dependent variable and the independent variables will be the previous experience of VTE (categorized as severe, moderate, or mild), previous experience with prophylactic LMWH (yes, problematic; yes, no problems; no prior experience), level of education (some postsecondary versus no postsecondary) and study site.

We will perform a standard multiple variable logistic regression to explore the determinants on the choice for or against LMWH prophylaxis in the real life scenario. The dependent variable will be the choice for or against LMWH prophylaxis and the independent variables will be the previous experience of VTE, previous experience with LMWH prophylaxis, level of education, study site, and high or low risk for recurrence.

A hierarchical logistic regression in which the dependent variable will be the choice to receive or not receive LMWH from the results of the hypothetical scenario exercise will also be undertaken. The independent variable within the first level will be the magnitude of the absolute risk reduction (high, moderate and low). The second level, nested within the absolute risk reduction will include previous experience of VTE, previous experience with LMWH, level of education and study site. Finally, we will perform a multiple variable linear regression with the threshold as the dependent variable and with utilities as independent variables. We will evaluate the interaction term of utilities and VTE in the regression model.

Using an independent sample *t*-test, we will evaluate if the responses between women who are pregnant or planning a pregnancy are different to those who are not.

The scatter plots of each of the participants’ utilities, as determined by the FT ratings of the health states, and the VTE thresholds will be examined to evaluate the relation between participant utilities and their VTE threshold. We will calculate Pearson’s correlations between each of the utilities for the health states and the threshold.

We believe that a lack of consistency in patient responses is the best way to detect a problem with individual patient understanding, which if it occurs frequently enough (i.e. > or = 25% of participants), may represent a problem in the way in which the information is being presented. Therefore, we will review consistency data after 10, 20, 30, and 40 women have been enrolled. We will also examine consistency data prior to these pre-specified points if there is concern about the level of inconsistency in patient responses. Table 4 contains frequencies of inconsistent responses, which would favor a review of the study scripts and presentation tools. We will also compare responses between women who are consistent and those who are inconsistent in their responses, even after they are provided with the opportunity to correct discrepancies in their responses. If the results differ between these groups, our primary analysis will include only those with consistent responses.

**Table 4:** Guidelines for frequencies of inconsistent responses favoring review of study scripts and presentation tools

**Frequency of inconsistent response**	**%**	**Lower bound of 95% CI**
> or = 5/10	50.0	23.7
> or = 8/20	40.0	21.9
> or = 10/30	33.3	19.2
> or = 12/40	30.0	18.1

For all key analyses above, we will compare results and the pattern of responses in women who answer the “understanding” questions correctly and those who do not. A similar comparison will also be made according to the interviewer’s impression of patient understanding. If the results differ between women categorized as understanding and not understanding, our primary analysis will focus on women with an apparent high level of understanding.

Finally, we will perform a “personalized decision analysis” for each woman in the study. We will update a prior decision analytic model examining prophylactic LMWH in pregnant women with a history of prior VTE (Figure 4) [87], using each women’s visual analog scale utility assessments for the relevant outcomes of DVT in pregnancy, PE in pregnancy, obstetrical hemorrhage and the burden of LWMH prophylaxis in pregnancy. Given that the risk of heparin-induced thrombocytopenia (<0.1%) and heparin associated osteoporosis (<1%) are either not increased or minimally increased with prophylactic LMWH [65,68], we will not consider these health states in the decision analysis. We will place the health state utilities in the decision model for each woman’s current situation and the three hypothetical scenarios. We will compare the decision model’s results with the VTE threshold selected in the probability trade-off tasks.

#### Sample size

Previous research from our group in patients with atrial fibrillation [83], and from other groups studying non-pregnant women with prior VTE [85], suggests that moderately precise estimates of patient preference can be obtained with sample sizes of approximately 100 participants. In the most relevant recent experience, co-investigators on the current project enrolled 96 patients with risk factors of atrial fibrillation [85]. The primary endpoint of that study was the threshold number of bleeds patients were willing to accept over 2 years given a reduction of 3 strokes over a 2-year period. This is directly analogous to the primary endpoint of our study, the VTE threshold. In the atrial fibrillation study, the mean number of bleeds that patients were willing to accept was 15. With a sample size of 96 patients, the confidence interval around the mean number of acceptable bleeds was 12 to 18, which we judged to be an acceptable range of uncertainty. The range of bleeds that patients chose varied over an extremely wide range (from 0 to 100). The entire range of possible VTE prevented for LWMH to be acceptable in the current study will range from 0 to 16, a much narrower range, which is certain to result in much smaller variability, and thus an appreciably smaller confidence interval. We are, thus, confident that our projected sample size of 100 will yield a sufficiently precise estimate of our primary outcome, the mean number of VTE prevented required to tolerate LMWH.

#### Safety and ethical considerations

This study does not pose any safety risks to participating women. Research Ethics Board approval has been obtained in Canada (Hamilton Health Sciences), Finland (The Coordinating Ethics Committee of Helsinki and Uusimaa Hospital District) and USA (Institutional Review Board of the State University of New York at Buffalo), and will be obtained from all other participating sites. All women will provide written informed consent prior to participating in this study.

## Discussion

Producing guidelines for management of pregnant women with prior VTE is problematic because there is little reliable information in the literature to guide the value and preference judgments necessary for making recommendations. Clinical management is challenging because of the lack of a standardized approach to presenting information and eliciting patients' choice. Our study will be the first to explore the preferences of women at risk of pregnancy-related VTE with respect to the use of antithrombotic therapy.

Our study has several strengths. We will address some of the limitations of the previous studies in the field of decision-making. These studies using direct choice methods, and in particular the probability trade-off, have shown many extreme choices that raise doubts about whether all patients understood the decisions they were making [84,88]. We pilot-tested standardized scripts that were reviewed by expert and non-expert clinicians, and allied health professionals to ensure appropriate readability and understandability. We are presenting probability information in three different ways. We have trained research assistants and will undertake quality control of the data as it accumulates.

We will check for consistency in the participants’ choice to either take or not take LMWH for a given probability of DVT. Participants will have discrepancies brought to their attention and will have the opportunity to change their responses. We will use standardized questions to test understanding of the information presented and the interviewer will provide a rating of the extent to which she or he believes the respondent had a clear understanding of the questions and their confidence in this assessment. Our analysis will include an exploration of whether patterns of responses differ (1) between those with an apparently high level of understanding and those who do not, (2) between those whose answers are consistent and those with inconsistent answers, and (3) between those who, according to the interviewer’s impression of patient understanding, show a correct understanding and those who do not. Finally, our multicenter international design limits the influence of local views about the use of LMWH prophylaxis for prevention of recurrent pregnancy-associated VTE and enhances generalizability.

Our study has some potential limitations. Our sample includes women not contemplating becoming pregnant. There are logistical challenges in prospectively identifying such a group and in ensuring their participation prior to their receiving other information about their choice. We believe that the inclusion of these women is a reasonable given the proximity to the necessity of a choice for these women and that their responses will reflect their true, thoughtful preferences. We will, nevertheless, evaluate whether the responses between women who are pregnant or planning a pregnancy are different to those who are not.

The research outlined in this protocol aims to provide explicit, quantitative expressions of women's valuations of health states related to recurrent VTE and its prevention with LMWH. This information will improve the quality of formal decision analyses evaluating whether the effectiveness of prophylaxis outweighs risks and burden of therapy and provide relevant information for guideline developers providing recommendations for clinical practice.

Our protocol addresses some of the limitations of the previous studies in the field of decision-making and describes innovative approaches to checking for understanding and systematically exploring inconsistencies. The methods described in this protocol are novel and rigorous, and could be utilized immediately by those working in the area of optimizing patients' medical decision-making.

## Competing interests

SMB has received honoraria from Leo Pharma Inc.; Sanofi-Aventis Canada and Pfizer Canada (all manufacturers of LMWH). The other authors declare no competing interests.

## Authors’ contributions

GHG, SMB and PA-C conceived the study. GHG, SMB, PA-C, SE, KAOT, ME and EA contributed to the study design. PA-C and SE completed the first draft of the manuscript and are co-first authors. All authors read, critically revised and approved the final manuscript to be published.

## Authors’ information

PA-C is a researcher in the Iberoamerican Cochrane Centre at the CIBERESP-IIB Sant Pau in Barcelona, Spain. SE is a doctoral student in the Department of Clinical Epidemiology and Biostatistics at McMaster University. GHG is a Professor in the Departments of Medicine and Clinical Epidemiology and Biostatistics at McMaster University. KAOT is a postdoctoral research fellow in the Department of Clinical Epidemiology and Biostatics at McMaster University and a urology resident in the Department of Urology, Helsinki University Central Hospital and University of Helsinki, Helsinki, Finland. IN is a graduate student in the Department of Clinical Epidemiology and Biostatistics at McMaster University and an Assistant Professor in the Department of Internal Medicine at Pontificia Universidad Católica de Chile. SDM is an Associate Professor in the Departments of Obstetrics & Gynecology, Clinical Epidemiology & Biostatics, and Radiology. EA is an Associate Professor in the Department of Medicine at SUNY in Buffalo, New York and a part-time Associate Professor in the Department of Clinical Epidemiology & Biostatistics at McMaster University. ME is a Professor of Clinical Medicine in the Division of General Internal Medicine and Center for Clinical Effectiveness at the University of Cincinnati in Cincinnati, Ohio. SMB is an Associate Professor in the Department of Medicine at McMaster University.

## Pre-publication history

The pre-publication history for this paper can be accessed here:

http://www.biomedcentral.com/1471-2393/12/40/prepub

## Supplementary Material

Additional file 1 **Appendix I.** Health States (1-4). **Appendix II.** Questions to Assess Understanding. Click here for file

## References

[B1] McColl MD Ellison J Greer IA Tait AC Walker ID Prevention of the post-thrombotic syndrome in young women with previous venous thromboembolism Br J Haematol 2000 108 272 274 10.1046/j.1365-2141.2000.01877.x 10691854

[B2] Lewis G Saving Mothers’ Lives: Reviewing maternal deaths to make motherhood safer – 2003–2005. Confidential Enquiry into Maternal and Child Health: The Sixth Report of the Confidential Enquiries into Maternal Death in the United Kingdom 2007 RCOG Press, London

[B3] Chang J Elam-Evans LD Berg CJ Herdon J Flowers L Seed KA Syverson CJ Pregnancy-related mortality surveillance- United States, 1991–1999 MMWR Surveill Summ 2003 52 1 88 12825542

[B4] James AH Jamison MG Brancazio LR Myers ER Venous thromboembolism during pregnancy and the postpartum period: incidence, risk factors, and mortality Am J Obstet Gynecol 2006 194 1311 1315 10.1016/j.ajog.2005.11.008 16647915

[B5] Weindling AM The confidential enquiry into maternal and child health (CEMACH) Arch Dis Child 2003 88 1034 1037 10.1136/adc.88.12.1034 14670760 PMC1719387

[B6] Pabinger I Grafenhofer H Kyrle PA Quehenberger P Mannhalter C Lechner K Kaider A Temporary increase in the risk for recurrence during pregnancy in women with a history of venous thromboembolism Blood 2002 100 1060 1062 10.1182/blood-2002-01-0149 12130523

[B7] Badaracco MA Vessey MP Recurrence of venous thromboembolic disease and use of oral contraceptives Br Med J 1974 1 215 217 10.1136/bmj.1.5901.215 4818161 PMC1633083

[B8] Tengborn L Bergqvist D Matzsch T Bergqvist A Hedner U Recurrent thromboembolism in pregnancy and puerperium. Is there a need for thromboprophylaxis? Am J Obstet gynecol 1989 160 90 94 2912109 10.1016/0002-9378(89)90095-1

[B9] Brill-Edwards P Ginsberg JS Gent M Hirsh J Burrows R Kearon C Geerts W Kovacs M Weitz JI Robinson KS Whittom R Couture G Recurrence of Clot in This Pregnancy Study Group Safety of withholding heparin in pregnant women with a history of venous thromboembolism. Recurrence Of Clot In This Pregnancy Study Group N Engl J Med 2000 343 1439 1444 10.1056/NEJM200011163432002 11078768

[B10] de Swiet M Floyd E Letsky E Low risk of recurrent thromboembolism in pregnancy Br J Hosp Med 1987 38 264 3676551

[B11] Howell R Fidler J Letsky E de Swiet M The risks of antenatal subcutaneous heparin prophylaxis: a controlled trial Br J Obstet Gynaecol 1983 90 1124 1128 10.1111/j.1471-0528.1983.tb06458.x 6360198

[B12] De Stefano V Martinelli I Rossi E Battaglioli T Za T Mannuccio Mannucci P Leone G The risk of recurrent venous thromboembolism in pregnancy and puerperium without antithrombotic prophylaxis Br J Haematol 2006 135 386 391 10.1111/j.1365-2141.2006.06317.x 16984390

[B13] Pabinger I Grafenhofer H Kaider A Kyrle PA Quehenberger P Mannhalter C Lechner K Risk of pregnancy-associated recurrent venous thromboembolism in women with a history of venous thromboembolism J Thromb Haemost 2005 3 949 954 10.1111/j.1538-7836.2005.01307.x 15869590

[B14] Baglin T Luddington R Brown K Baglin C Incidence of recurrent venous thromboembolism in relation to clinical and thrombophilic risk factors: prospective cohort study Lancet 2003 362 523 526 10.1016/S0140-6736(03)14111-6 12932383

[B15] Christiansen SC Cannegieter SC Koster T Vandenbroucke JP Rosendaal FR Thrombophilia, clinical factors, and recurrent venous thrombotic events JAMA 2005 293 2352 2361 10.1001/jama.293.19.2352 15900005

[B16] Hansson PO Sorbo J Eriksson H Recurrent venous thromboembolism after deep vein thrombosis: incidence and risk factors Arch Intern Med 2000 160 769 774 10.1001/archinte.160.6.769 10737276

[B17] Heit JA Mohr DN Silverstein MD Petterson TM O'Fallon WM Melton LJ 3rd Predictors of recurrence after deep vein thrombosis and pulmonary embolism: a population-based cohort study Arch Intern Med 2000 160 761 768 10.1001/archinte.160.6.761 10737275

[B18] Iori A Kearon C Filippuci E Marcucci M Macura A Pengo V Siragusa S Palareti G Risk of recurrence after a first episode of symptomatic venous thromboembolism provoked by a transient risk factor: a systematic review Arch intern Med 2010 170 1710 1716 10.1001/archinternmed.2010.367 20975016

[B19] Kearon C Lopez JA, Kearon C, Lee AYY Risk factors for recurrent venous thromboembolism and their implications for treatment Deep venous thrombosis 2004 Hematology (Am Hem Soc Hematol Educ Program) 439 456 10.1182/asheducation-2004.1.439 15561697

[B20] Kearon C Ginsberg JS Anderson DR Kearon C Ginsberg JS Anderson DR Kovacs MJ Wells P Julian JA Mackinnon B Demers C Douketis J Turpie AG Van Nguyen P Green D Kassis J Kahn SR Solymoss S Desjardins L Geerts W Johnston M Weitz JI Hirsh J Gent M for the SOFAST Investigators Comparison of 1 month with 3 months of anticoagulation for a first episode of venous thromboembolism associated with a transient risk factor J Thromb Haemost 2004 2 743 749 10.1046/j.1538-7836.2004.00698.x 15099280

[B21] Levine MN Hirsh J Gent M Turpie AG Weitz J Ginsberg J Geerts W LeClerc J Neemeh J Powers P Optimal duration of oral anticoagulant therapy: a randomized trial comparing four weeks with three months of warfarin in patients with proximal deep vein thrombosis Thromb Haemost 1995 74 606 611 8584992

[B22] Pinede L Ninet J Duhaut P Chabaud S Demolombe-Rague S Durieu I Nony P Sanson C Boissel JP for the DOTAVK Study Comparison of 3 and 6 months of anticoagulant therapy after a first episode of proximal deep vein thrombosis or pulmonary embolism and comparison of 6 and 12 weeks of therapy after isolated calf deep vein thrombosis Circulation 2001 103 2453 2460 10.1161/01.CIR.103.20.2453 11369685

[B23] Prandoni P Lensing AWA Buller HR Cogo A Prins MH Cattelan AM Cuppini S Noventa F ten Cate JW Deep vein thrombosis and the incidence of subsequent symptomatic cancer N Engl J Med 1992 327 1128 1133 10.1056/NEJM199210153271604 1528208

[B24] Research Committee of the British Thoracic Society Optimum duration of anticoagulation for deep vein thrombosis and pulmonary embolism Lancet 1992 340 873 876 1357297

[B25] Schulman S Rhedin A-S Lindmarker P Carlsson A Lärfars G Nicol P Loogna E Svensson E Ljungberg B Walter H for the Duration of Anticoagulation Trial Study Group A comparison of six weeks with six months of oral anticoagulant therapy after a first episode of venous thromboembolism N Engl J Med 1995 332 1661 1665 10.1056/NEJM199506223322501 7760866

[B26] Agnelli G Prandoni P Santamaria MG Bagatella P Iorio A Bazzan M Moia M Guazzaloca G Bertoldi A Tomasi C Scannapieco G Ageno W for the Warfarin Optimal Duration Italian Trial Investigators Three months versus one year of oral anticoagulant therapy for idiopathic deep vein thrombosis N Engl J Med 2001 345 165 169 10.1056/NEJM200107193450302 11463010

[B27] Agnelli G Prandoni P Becattini C Silingardi M Taliani MR Miccio M Imberti D Poggio R Ageno W Pogliani E Porro F Zonzin P for the Warfarin Optimal Duration Italian Trial Investigators Extended oral anticoagulant therapy after a first episode of pulmonary embolism Ann Intern Med 2003 130 19 25 12834314 10.7326/0003-4819-139-1-200307010-00008

[B28] Campbell A Bentley DP Prescott RJ Routledge PA Shetty HGM Williamson IJ Anticoagulation for three months versus six months in patients with deep vein thrombosis or pulmonary embolism or both: randomised trial BMJ 2007 334 674 680 10.1136/bmj.39098.583356.55 17289685 PMC1839169

[B29] Kearon C Gent M Hirsh J Weitz J Kovacs MJ Anderson DR Turpie AG Green D Ginsberg JS Wells P MacKinnon B Julian JA A comparison of three months of anticoagulation with extended anticoagulation for a first episode of idiopathic venous thromboembolism N Engl J Med 1999 340 901 907 10.1056/NEJM199903253401201 10089183

[B30] Ridker PM Goldhaber SZ Danielson E Rosenberg Y Eby CS Deitcher SR Cushman M Moll S Kessler CM Elliott CG Paulson R Wong T Bauer KA Schwartz BA Miletich JP Bounameaux H Glynn RJ Long-term low-intensity warfarin therapy for prevention of recurrent venous thromboembolism N Engl J Med 2003 348 2453 2460 10.1056/NEJMe030081 12601075

[B31] Eichinger S Pabinger I Stumpflen A Hirschl M Bialonczyk C Schneider B Mannhalter C Minar E Lechner K Kyrle PA The risk of recurrent venous thromboembolism in patients with and without factor V Leiden Thromb Haemost 1997 77 624 628 9134632

[B32] Eichinger S Minar E Hirschl M Bialonczyk C Stain M Mannhalter C Stümpflen A Schneider B Lechner K Kyrle PA The risk of early recurrent venous thromboembolism after oral anticoagulant therapy in patients with the G20210A transition in the prothrombin gene Thromb Haemost 1999 81 14 17 10348706

[B33] Ho WK Hankey GJ Quinlan DJ Eikelboom JW Risk of recurrent venous thromboembolism in patients with common thrombophilia: a systematic review Arch Intern Med 2006 166 729 736 10.1001/archinte.166.7.729 16606808

[B34] Lindmarker P Schulman S Sten-Linder M Wiman B Egberg N Johnsson H The risk of recurrent venous thromboembolism in carriers and noncarriers of the G1961A allele in the coagulation factor V gene and the G20210A allele in the prothrombin gene Thromb Haemost 1999 81 684 689 10365737

[B35] Middeldorp S Weitz JI, Middeldorp S, Geerts W, Heit JA Thrombophilic defects Thrombophilia and new anticoagulant drugs 2004 Hematology (Am Hem Soc Hematol Educ Program) 424 438 10.1182/asheducation-2004.1.424 15561696

[B36] Miles JS Miletich JP Goldhaber SZ Hennekens CH Ridker PM G20210A mutation in the prothrombin gene and the risk of recurrent venous thromboembolism J Am Coll Cardiol 2001 37 215 218 10.1016/S0735-1097(00)01080-9 11153741

[B37] Palareti G Legnani C Cosmi B Valdré L Lunghi B Bernardi F Coccheri S Predictive value of d-dimer test for recurrent venous thromboembolism after anticoagulation withdrawal in subjects with a previous diopathic event and in carriers of congenital thrombophilia Circulation 2003 108 313 318 10.1161/01.CIR.0000079162.69615.0F 12847064

[B38] Ridker PM Miletich JP Stampfer MJ Goldhaber SZ Lindpaintner K Hennekens CH Factor V Leiden and risks of recurrent idiopathic venous thromboembolism Circulation 1995 92 2800 2802 10.1161/01.CIR.92.10.2800 7586244

[B39] Schulman S Lindmarker P Holmstrom M Lärfars G Carlsson A Nicol P Svensson E Ljungberg B Viering S Nordlander S Leijd B Jahed K Hjorth M Linder O Beckman M Postthrombotic syndrome, recurrence, and death 10 years after the first episode of venous thromboembolism treated with warfarin for 6 weeks or 6 months J Thromb Haemost 2006 4 734 742 10.1111/j.1538-7836.2006.01795.x 16634738

[B40] Simioni P Prandoni P Lensing AW Manfrin D Tormene D Gavasso S Girolami B Sardella C Prins M Girolami A Risk for subsequent venous thromboembolic complications in carriers of the prothrombin or the factor V gene mutation with a first episode of deep vein thrombosis Blood 2000 96 3329 3333 11071624

[B41] Chan W Anand S Ginsberg JS Anticoagulation of pregnant women with mechanical heart valves: A systematic review of the literature Arch Intern Med 2000 160 191 196 10.1001/archinte.160.2.191 10647757

[B42] Ginsberg J Hirsh J Turner CD Levine MN Burrows R Risk to the fetus of anticoagulant therapy during pregnancy Thromb Haemost 1989 61 197 203 2665171

[B43] Hall J Paul RM Wilson KM Maternal and fetal sequelae of anticoagulation during pregnancy Am J Med 1980 68 122 140 10.1016/0002-9343(80)90181-3 6985765

[B44] Hassouna A Allam H Anticoagulation of pregnant women with mechanical heart valve prosthesis: a systematic review of the literature (2000–2009) J Coagul Disorders 2010 2 81 88

[B45] Forestier F Daffos F Capella-Pavlovsky M Low molecular-weight heparin (PK 10169) does not cross the placenta during the second trimester of pregnancy, study by direct fetal blood sampling under ultrasound Thromb Res 1984 34 557 560 10.1016/0049-3848(84)90260-3 6740572

[B46] Bates S Greer IA Pabinger I Sofaer S Hirsh J Venous thromboembolism, thrombophilia, antithrombotic therapy and pregnancy: American College of Chest Physicians Evidence-Based Clinical Practice Guidelines (8the Edition). The Eighth ACCP Conference on Antithrombotic and Thrombolytic Therapy Chest 2008 133 844S 886S 10.1378/chest.08-0761 18574280

[B47] Greer I Nelson-Piercy C Low-molecular-weight heparins for thromboprophylaxis and treatment of venous thromboembolism in pregnancy: a systematic review of safety and efficacy Blood 2005 106 401 407 10.1182/blood-2005-02-0626 15811953

[B48] Lepercq J Conard J Borel Derlon A Darmon JY Boudignat O Francoual C Priollet P Cohen C Yvelin N Schved JF Tournaire M Borg JY Venous thromboembolism during pregnancy: a retrospective study of enoxaparin safety in 624 pregnancies BJOG 2001 108 1134 1140 11762651 10.1111/j.1471-0528.2003.00272.x

[B49] Rodie V Thomson AJ Stewart FM Quinn AJ Walker ID Greer IA Low-molecular-weight heparin for treatment of venous thromboembolism in pregnancy: a case series BJOG 2002 109 1020 1024 10.1111/j.1471-0528.2002.01525.x 12269676

[B50] Rowan J McClintock C Taylor RS North RA Prophylactic and therapeutic enoxaparin: indications, outcomes and monitoring Aust NZ J Obstet Gynaecol 2003 43 123 128 10.1046/j.0004-8666.2003.00034.x 14712967

[B51] Sanson B Lensing AW Prins MH Ginsberg JS Barkagan ZS Lavenne-Pardonge E Brenner B Dulitzky M Nielsen JD Boda Z Turi S Mac Gillavry MR Hamulyák K Theunissen IM Hunt BJ Büller HR Safety of low-molecular-weight heparin in pregnancy: a systematic review Thromb Haemost 1999 81 668 672 10365733

[B52] Smith M Norris LA Steer PJ Savidge GF Bonnar J Tinzaparin sodium for thrombosis treatment and prevention during pregnancy Am J Obstet Gynecol 2004 190 495 501 10.1016/S0002-9378(03)00953-0 14981396

[B53] Sorensen H Johnsen SP Larsen H Pedersen L Nielsen GL Møller M Birth outcomes in pregnant women treated with low-molecular-weight heparin Acta Obstet Gynecol Scan 2000 79 655 659 10949230

[B54] Ulander V Stenqvist P Kaaja R Treatment of deep venous thrombosis with low-molecular-weight heparin during pregnancy Thromb Res 2002 106 13 17 10.1016/S0049-3848(02)00074-9 12165283

[B55] Badawy A Khiary M Sherif LS Hassan M Ragab A Abdelall I Low molecular weight heparin in patients with recurrent early miscarriages of unknown aetiology J Obstet Gynaecol 2008 28 280 284 10.1080/01443610802042688 18569468

[B56] Brenner B Hoffman R Carp H Dulitsky M Younis J for the LIVE-ENOX Investigators Efficacy and safety of two doses of enoxaparin in women with thrombophilia and recurrent pregnancy loss: the LIVE-ENOX study J Thromb Haemost 2005 3 227 229 10.1111/j.1538-7836.2004.01090.x 15670024

[B57] Clark P Walker ID Langhorne P Crichton L Thomson A Greaves M Whyte S Greer IA on behalf of the Scottish Pregnancy Intervention Study (SPIN) collaborators SPIN (Scottish Pregnancy Intervention) study: a multicenter, randomized controlled trial of low molecular weight heparin and low dose aspirin in women with recurrent miscarriage Blood 2010 115 4162 4167 10.1182/blood-2010-01-267252 20237316

[B58] Dargaud Y Rugeri L Ninet J Negrier C Treciak MC Management of pregnant women with increaed risk of venous thrombosis Int J Gynecol Obstet 2004 90 203 207 10.1016/j.ijgo.2005.05.003 15964002

[B59] Darguad Y Rugeri L Vergnes MC Arnuti B Miranda P Negrier C Bestion A Desmurs-Clavel H Ninet J Gaucherand P Rudigoz RC Berland M Champion F Trzeciak MC A risk score for the management of pregnant women with increased risk of venous thromboembolism: a multicentre prospective study Br J Haematol 2009 145 825 835 10.1111/j.1365-2141.2009.07698.x 19388925

[B60] Dolitzky M Inbal A Segal Y Weiss A Brenner B Carp H A randomized study of thromboprophylaxis in women with unexplained consecutive recurrent miscarriages Fertil Steril 2006 86 362 366 10.1016/j.fertnstert.2005.12.068 16769056

[B61] Fawzy M Shokeir T El-Tatongy M Warda O El-Refaiey A-A A Mosbah A Treatment options and pregnancy outcome in women with idiopathic recurrent miscarriage: a randomized placebo-controlled study Arch Gynecol Obstet 2008 278 33 38 10.1007/s00404-007-0527-x 18071727

[B62] Gris J-C Mercier E Quere I Lavigne-Lissalde G Cochery-Nouvellon E Hoffet M Ripart-Neveu S Tailland M-L Dauzat M Mares P Low molecular weight heparin versus low dose aspirin in women with one fetal loss and a constitutional thrombophic disorder Blood 2004 103 3695 3699 10.1182/blood-2003-12-4250 14739212

[B63] Gris J-C Chauleur C Faillie J-L Baer G Mares P Fabbro-Peray P Quere I Lefrant J-Y Haddad B Dauzat M Enoxaparin for the secondary prevention of placental vascular complications in women with abruption placentae Thromb Haemost 2010 104 771 779 10.1160/TH10-03-0167 20694277

[B64] Kaandorp S Goddijn M van der Post JAM Hutten BA Verhoeve HR Hamulyak K Mol BW Folkeringa N Nahuis M Papatsonis DNM Buller HR van der Veen F Middeldorp S Aspirin plus heparin or aspirin alone in women with recurrent miscarriage N Engl J Med 2010 362 1586 1596 10.1056/NEJMoa1000641 20335572

[B65] Le Templier G Rodger MA Heparin-induced osteoporosis and pregnancy Curr Opin Pulm Med 2008 14 403 407 10.1097/MCP.0b013e3283061191 18664969

[B66] Noble L Kutteh WH Lashey N Franklin RD Herrada J Antiphospholipid antibodies associated with recurrent pregnancy loss: prospective, multicenter, controlled pilot study comparing treatment with low molecular weight heparin versus unfractionated heparin Fertil Steril 2005 83 684 690 10.1016/j.fertnstert.2004.11.002 15749498

[B67] Regy E Garneau P David M Gautheir R Leduc L Michon N Morin F Demers C Kahn SR Magee LA Rodger M Dalteparin for the prevention of recurrence of placental-mediated complications of pregnancy in women without thrombophilia: a pilot randomized controlled trial J Thromb Haemost 2009 7 58 64 10.1111/j.1538-7836.2008.03230.x 19036070

[B68] Warkentin T Greinacher A Koster A Lincoff M Treatment and prevention of heparin-induced thrombocytopenia: American College of Chest Physicians Evidence-Based Clinical Practice Guidelines (8th Edition) Chest 2008 133 Suppl 340S 380S 10.1378/chest.08-0677 18574270

[B69] Tooher R Gates S Dowswell T Davis LJ Prophylaxis for venous thromboembolic disease in pregnancy and the early postnatal period Cochrane Database of Systematic Reviews 2010 Issue 5. Art. No.: CD001689 10.1002/14651858.CD001689.pub2 PMC4175551 20464719

[B70] Gates S Brocklehurst P Ayers S Bowler U for the Thromboprophylaxis in Pregnancy Advisory Group Thromboprophylaxis and pregnancy: two randomized controlled pilot trials that used low-molecular-weight heparin Am J Obstet Gynecol 2004 191 1296 1303 10.1016/j.ajog.2004.03.039 15507957

[B71] Geerts W Bergqvist D Pineo GF Heit JA Samama CM Lassen MR Colwell CW Prevention of venous thromboembolism: American College of Chest Physicians Evidence-Based Clinical Practice Guidelines (Eighth Edition). The Eighth ACCP Conference on Antithrombotic and Thrombolytic Therapy Chest 2008 133 381S 453S 10.1378/chest.08-0656 18574271

[B72] Man-Son-Hing M Gage BF Montgomery AA Howitt A Thomson R Devereaux PJ Protheroe J Fahey T Armstrong D Laupacis A Preference-Based Antithrombotic Therapy in Atrial Fibrillation: Implications for Clinical Decision Making Medical Decision Making 2005 25 548 559 10.1177/0272989X05280558 16160210

[B73] Llewellyn-Thomas H Williams JI Levy L Naylor CD Using a trade-off technique to assess patients’ treatment preferences for benign prostatic hyperplasia Med Decis Making 1996 16 262 272 10.1177/0272989X9601600311 8818125

[B74] Llewellyn-Thomas H Investigating patients’ preferences for different treatment options Can J Nurs Res 1997 29 45 64 9505582

[B75] Green C Brazier J Deverill M Valuing health-related quality of life: a review of health state valuation techniques Pharmacoeconomics 2000 17 151 165 10.2165/00019053-200017020-00004 10947338

[B76] Torrance G Measurement of health state utilities for economic appraisal J Health Econ 1986 5 1 30 10.1016/0167-6296(86)90020-2 10311607

[B77] von Neuman J Morgenstern O Theory of Games and Economic Behaviour (3rd ed) 1953 3 Princeton University Press, Princeton, MJ

[B78] Puhan M Guyatt G Montori V Devereaux PJ Bhandari M Griffith L Goldstein R Schünemann HJ The standard gamble demonstrated lower reliability than the feeling thermometer J Clin Epidemiol 2005 58 458 465 10.1016/j.jclinepi.2004.07.010 15845332

[B79] Schünemann H Guyatt GH Griffith L Stubbing D Goldstein R A clinical trial to evaluate the responsiveness and validity of two direct health state preference instruments administered with and without hypothetical marker states in chronic respiratory disease Medical Decision Making 2003 23 140 149 10.1177/0272989X03251243 12693876

[B80] Schünemann H Armstrong D Fallone C Barkun A Degli’Innocenti A Heels-Ansdell D Wiklund I Tanser L Chiba N Austin P Van Zanten S El-Dika S Guyatt GH A randomized multi-center trial to evaluate simple utility elicitation techniques in patients with gastroesophageal reflux disease Med Care 2004 42 32 42 10.1097/00005650-200411000-00013 15586841

[B81] Schünemann H Goldstein R Mador J McKim D Stahl E Griffith L Bayoumi A Austin P Guyatt GH Do clinical marker states improve responsiveness and construct validity of the standard gamble and feeling thermometer: a randomized multi-center trial in patients with chronic respiratory disease Qual Life Res 2006 14 1 14 10.1007/s11136-005-0126-x 16411026

[B82] Schünemann H Norman G Puhan M Griffith L Wiklund I Heels-Andell D Montori VM Goldstein R Mador JM Guyatt GH Application of generalizability theory confirmed lower reliability of the standard gamble than the feeling thermometer J Clin Epidemiol 2007 60 1256 1262 10.1016/j.jclinepi.2007.03.010 17998080

[B83] Alonso-Coello P Montori VM Sola I Schunemann HJ Devereaux PJ Charles C Roura M Díaz MG Souto JC Alonso R Oliver S Ruiz R Coll-Vinent B Diez AI Gich I Guyatt G Values and preferences in oral anticoagulation in patients with atrial fibrillation, physicians’ and patients’ perspectives: protocol for a two-phase study BMC Health Services Res 2008 8 221 234 10.1186/1472-6963-8-221 PMC2613147 18954427

[B84] Devereaux P Anderson DR Garner MJ Differences between perspectives of physicians and patients on anticoagulation in patients with atrial fibrillation: observational study BMJ 2001 232 1211 1218 10.1136/bmj.323.7323.1218 PMC59994 11719412

[B85] Locadia M Bossuyt PMM Stalmeirer PFM Sprangers MAG van Dongen CJJ Middeldorp S Bank I van der Meer J Hamulyák K Prins MH Treatment of venous thromboembolism with vitamin K antagonists: patient’ health valuations and treatment preferences Thromb Haemost 2004 92 1336 1341 15583742 10.1160/TH04-02-0075

[B86] MacLean S Evidence Based Medicine and Patient Choice: Recent Progress and Persisting Challenges 2010 McMaster University, Hamilton, ON

[B87] Johnston J Brill-Edwards P Ginsberg JS Pauker SG Eckman MH Cost-effectiveness of prophylactic low molecular weight heparin in pregnant women with a prior history of venous thromboembolism Am J Med 2005 118 503 514 10.1016/j.amjmed.2004.12.009 15866253

[B88] Tversky A Kahneman D Judgment under uncertainty: heuristics and biases Science 1974 185 1124 1130 10.1126/science.185.4157.1124 17835457

