# Supplementary material for: Evaluating patient values and preferences for thromboprophylaxis decision making during pregnancy: a study protocol
Source: BMC Pregnancy Childbirth. 2012 May 30;12:40. doi: 10.1186/1471-2393-12-40 (PMC3495041; doi:10.1186/1471-2393-12-40)
Supplement: Additional file 1 — Appendix I. Health States (1-4). Appendix II. Questions to Assess Understanding. [file 1471-2393-12-40-S1.docx]

Additional File 1

Appendix I: Health States (1-4)

Appendix II: Questions to Assess Understanding

**Appendix I: Health States**

Box 1) Health state for the burden associated with LMWH prophylaxis

| **BAD THINGS ABOUT TAKING LOW MOLEcULAR WEIGHT HEPARIN to Prevent blood clots DUring pregnancy** | |
| --- | --- |
| **Preventative Blood Thinning** | - You use low molecular weight heparin needles beneath the skin for the rest of your pregnancy. - You or a family member learn to give these needles. - You continue blood thinners for at least 6 weeks after your baby is born, either with needles or with a tablet. If you choose the tablet, you will need to have blood tests on a regular basis to make sure you are using the right dose. |
| **Risks & Inconvenience** | - Your daily low molecular weight heparin needles sting. You get a bruise at the place where you put in the needle. - Taking needles might make you feel frightened. - If you get a rash with the injections, you may need to use a different type of heparin. - Because you are using low molecular weight heparin, your doctor will need to make special plans for your delivery date. If you go into labour when your blood is thinned, you may not be able to use the best way to reduce the pain of labour (a freezing needle in your back or epidural) and you may have a higher risk of bleeding. To prevent this, your delivery will be planned (also called an induction). If you go into labour early, your epidural may be delayed or you may not be able to receive one at all. - Using low molecular weight heparin to prevent blood clots may cause serious bleeding, thinning of the bones (osteoporosis), or an allergic reaction to heparin called heparin-induced thrombocytopenia or it may not cause these problems, doctors are not sure. If low molecular weight heparin does cause these problems, they are very rare. If they happen at all, it is likely to happen in less than 1 in 100 or 1% of pregnant women. |
| **Long-term Consequences** | - There are no long-term risks from taking low molecular weight heparin during your pregnancy. |

Box 2) Health state for the burden associated with pregnancy-related DVT and its treatment

| **PREGNANCY-ASSOCIATED BLOOD CLOT IN LEG** | |
| --- | --- |
| **Symptoms & Signs** | - Your leg hurts and it swells. It hurts more if you go for more than a short walk. |
| **Diagnosis & Treatment** | - Your doctor does an ultrasound test that shows that you have a blood clot leg. You stay in the Emergency Department overnight. - You worry about the bad things that may happen to your baby because of this blood clot. - Your doctor treats you with blood thinning needles of low molecular weight heparin beneath your skin each day. You or a family member learns to give these needles. - Treatment of your blood clot goes on for your whole pregnancy and for at least 6 weeks after you have your baby. - After you have your baby, your doctor might give you the same needle or switch you to a tablet. If you use the tablet you will have to travel for regular blood tests. |
| **Risks & Inconvenience** | - The needles sting. You bruise at the place where you put in the needle. - You may get skin problems like itching or an itchy rash. If you get these problems, you may have to use a different type of heparin. - Even though your doctor tells you that your baby is safe, you are worried that these blood thinning needles may not be safe for your baby. - You doctor tells you that there may be a small increase in the risk of serious bleeding, thinning of the bones (osteoporosis) and having an allergic reaction to heparin called heparin-induced thrombocytopenia. If there is a risk, it is very small. - Because you are using low molecular weight heparin, your doctor will need to make special plans for your delivery. If you go into labour when your blood is thinned, you may not be able to use the best way to reduce the pain of labour (a freezing needle in your back or epidural) and you may have a higher risk of bleeding. To prevent this, your delivery will be planned (also called an induction). If you go into labour early, your epidural may be delayed or you may not be able to receive one at all. |
| **Long-term Consequences** | - There are no problems for your baby from the blood clot or from the low molecular weight heparin. - Your leg goes back to normal. After needles or tables are stopped, you feel worried sometimes if you have pains in your leg. - You have a higher risk of blood clots in the future (your risk may be 3 to 5% in the first year after you stop treatment; lower after that). - Your doctor asks you if you would like to be tested for a clotting disorder. - Your doctor tells you that you may have to take low molecular weight heparin needles if you get pregnant again. |

Box 3) Health state for the burden associated with pregnancy-related PE and its treatment

| **PREGNANCY-ASSOCIATED BLOOD CLOT IN THE LUNGS** | |
| --- | --- |
| **Symptoms & Signs** | - For the past 3 days you find it hard to breathe while sitting. You have to rest if you climb stairs or walk outside of your home. - You have pain in your chest when you take a breath. - You feel very worried about your health and your baby. |
| **Diagnosis & Treatment** | - You have a test. When you have the test, you get a small dose of radiation. The radiation probably does not have any risk for you or your baby. If there is any risk, it is very small. The test tells your doctor that you have a blood clot in your lungs. You have to stay in hospital for several days. - You worry about the effects this test and your blood clot might have on your baby. - At first, your doctor treats you with blood thinners in your veins. After that you use needles of low molecular weight heparin. You take these needles beneath your skin every day for the rest of your pregnancy. You or a family member learns to give these needles. - Treatment of your blood clot goes on for your whole pregnancy and for at least 6 weeks after you have your baby. - After you have your baby, your doctor might give you the same needles or switch you to a tablet. If you take the tablet, you will have to travel for regular blood tests. |
| **Risks & Inconvenience** | - The needles sting. You bruise at the place where you put in the needle. - You may get skin problems like itching or an itch rash. If you get these problems, you may have to use a different type of heparin. - Even though your doctor tells you that your baby is safe, you are worried that these blood thinning needles may not be safe for your baby. - Your doctor tells you that there may be a small increase in the risk of serious bleeding, thinning of the bones (osteoporosis), and having an allergic reaction to heparin called heparin-induced thrombocytopenia. If there is a risk, it is very small. - Because you are using low molecular weight heparin, your doctor will need to make special plans for your delivery. If you go into labour when your blood is thinned, you may not be able to use the best way to reduce the pain of labour (a freezing needle in your back or epidural) and you may have a higher risk of bleeding. To prevent this, your delivery will be planned (also called an induction). If you go into labour early, your epidural may be delayed or you may not be able to receive one at all. |
| **Long-term Consequences** | - There are no problems for your baby is not affected from the blood clot or from the low molecular weight heparin needles. - Your breathing goes back to normal. After the needles or tablets are stopped, you feel worried sometimes if you have a pain in your chest or if you find it hard to breathe. - You have a higher risk of blood clots in the future (your risk may be 3 to 5% in the first year after stopping treatment; lower after that). - Your doctor asks you if you would like to be tested for a clotting disorder. - Your doctor tells you that you may have to take low molecular weight heparin needles if you get pregnant again. |

Box 4) Health state for the burden associated with major antepartum obstetrical hemorrhage and its treatment

| **MAJOR ANTEPARTUM OBSTETRICAL BLEED** | |
| --- | --- |
| **Symptoms & Signs** | - You start to have pain in your abdomen, contractions and bleeding from your vagina in the later part of your pregnancy. |
| **Treatment** | - You have to stay in the hospital. - Your doctor places a needle in your vein. You get fluids through this needle. - Your stop taking your low molecular weight heparin. - You have more blood tests. - The doctor does an ultrasound to see how your baby is doing. - Your doctor does a test to see if you baby’s heart is beating the way it should. - You stay in hospital for two or three days until your doctors are sure that your bleeding has slowed down or stopped. - Your doctor will see you frequently after you go home from the hospital. |
| **Recovery** | - You are worried about your baby's health. - You may deliver early and may be more likely to need a caesarean section. |
| **Long-term Consequences** | - Your baby is okay. - You feel worried with future pregnancies. - When you get pregnant again, your risk of similar bleeding problems is greater than if you had never had the bleeding problem. - In any future pregnancies, you will need to visit your doctor more often than if you had never had the bleeding problem. |

**Appendix II**

**Questions to Assess Understanding**

**[1] Looking at these 3 different imaginary situations or scenarios {*show the 3 Hypothetical Scenarios*}, what is the highest risk you can have for developing a blood clot if you do not take low molecular weight heparin during pregnancy?**

Is it:

____ 16%

____ 10%

____ 4%

____ Other: please write number

Thank-you for answering that question.

*For the interviewer: If the woman points to one scenario, it is enough. She doesn´t have to necessarily to calculate the actual value (percentage)*

**[2] Does the use of low molecular weight heparin increase or decrease the risk of a blood clot during pregnancy:**

_____ Increase

or

_____ Decrease

Thank-you for answering that question.

*For interviewer to complete based on the answers of the above questions*

*Please rate your own impression of the patient’s understanding on the seven point scale by circling a number. 1 represents “there is a major problem in understanding” and 7 represents “the patient understood perfectly”*

1 2 3 4 5 6 7

Major Problem Understood Perfectly
